# Buschke-Löwenstein Tumour: Successful Treatment with Minimally Invasive Techniques

**DOI:** 10.1155/2015/651703

**Published:** 2015-08-31

**Authors:** Estefânia Correia, António Santos

**Affiliations:** ^1^Family Practice Unit of Pedras Rubras, Rua Divino Salvador de Moreira 160, 4470-105 Maia, Portugal; ^2^Department of Dermatology, Portuguese Institute of Oncology, Portugal

## Abstract

We report a case of an 80-year-old female who presented with a four-year history of a growing mass in the perianal area with pain and bleeding during defaecation. Clinical examination revealed a locally destructive, cauliflower-like, verrucous mass measuring 10 × 12 cm in diameter. Histologic findings revealed a moderate degree of dysplasia of the epithelium with koilocytosis atypia, acanthosis, and parakeratosis, features that are consistent with Buschke-Löwenstein tumour. Polymerase-chain-reaction assay for human papillomavirus (HPV) showed an infection with HPV type 11. Full-thickness excision of involved skin was undertaken by cryotherapy and electrocautery over five months. The entire wound was left open to heal by secondary intention. After 3 years of follow-up, the patient has not experienced a recurrence, with excellent functional results, but the cosmetic results were satisfactory. These minimally invasive techniques can be safer and more cost-effective than surgery and the General Practitioner can play a key role in diagnosis.

## 1. Introduction

Buschke-Löwenstein tumour (BLT), also known as giant condyloma acuminatum, is a very rare, sexually transmitted disease that affects the anogenital region [1–3].

The human papillomavirus (HPV) has been identified as an important contributory factor in the development of BLT [4].

Although this is a well-differentiated, benign lesion, its management is often challenging due to the size, probable local invasion, and elevated recurrence rates [4].

Without a well-defined treatment protocol for BLT, many medical and/or surgical treatment options can be found in the literature with very different results [5].

We present a case report from the Portuguese Institute of Oncology and a review of the literature.

## 2. Case Report

**Figure 1:**
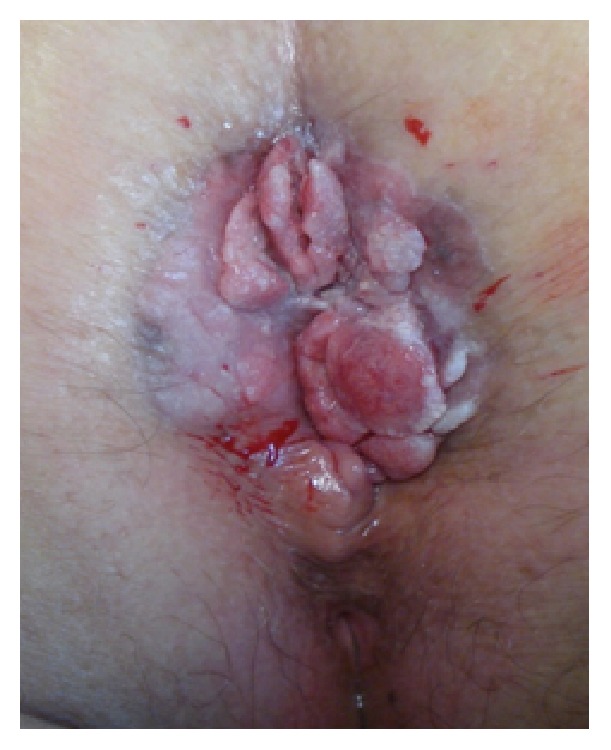
Large cauliflower-like giant condyloma acuminatum on the anogenital area.

An 80-year-old female was referred to the department of dermatology with a four-year history of a growing mass in the perianal area with pain and bleeding during defaecation. She had not received any treatment previously. The patient's medical history was as follows: cervical cancer diagnosed 25 years before, treated successfully with radiotherapy, and a non-Hodgkin lymphoma diagnosed 11 years before, treated with chemotherapy for one year. She denied sexual promiscuity and alcohol or drugs abuse. On dermatological examination, there was a cauliflower-like growth on her anogenital area, which was fleshy, sessile, and slightly friable in areas with some bleeding and foul-smelling, purulent exudate on the surface. Grossly, the lesion measured 10 × 12 cm with a central thickness of 3.5 cm (Figure 1).

**Figure 2:**
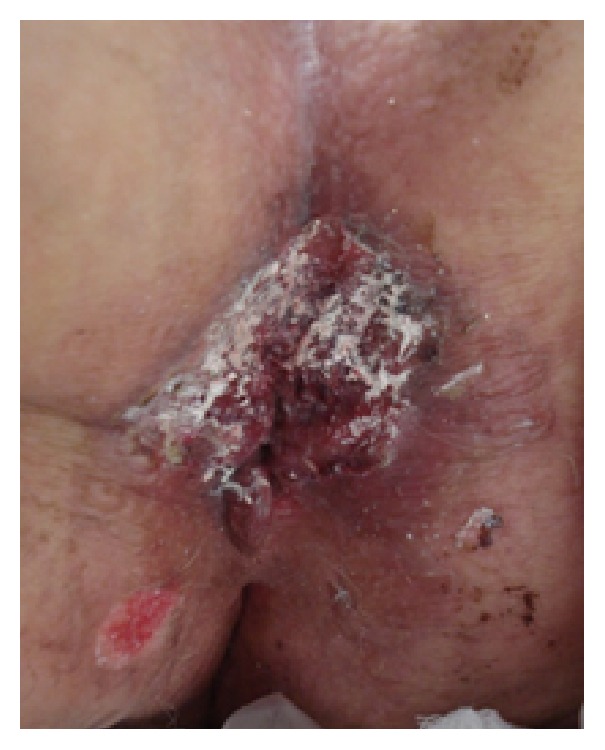
Perineum after electrocautery and cryotherapy.

Anoscopy with a flexible endoscope, vaginal speculum examination, and inguinal lymph node palpation were normal. Routine laboratory examinations and serological tests for syphilis, HIV, hepatitis B, and hepatitis C were negative. A thoracic-abdominal-pelvic computed tomography (CT) scan showed no local invasion and HPV-11 was found on large and deep biopsy specimen with polymerase chain reaction (PCR). The biopsy also showed moderate degree of dysplasia of the epithelium with koilocytosis atypia, acanthosis, and parakeratosis. Although surgical excision was recommended, the patient refused this due to its cosmetic and functional disability. After obtaining written consent, she underwent local excision using electrosurgery (200–300 volts) and cryotherapy (−50°C; 30 seconds) with liquid nitrogen. Two cycles of electrosurgery and three cycles of cryotherapy were administered over five months (Figure 2).

**Figure 3:**
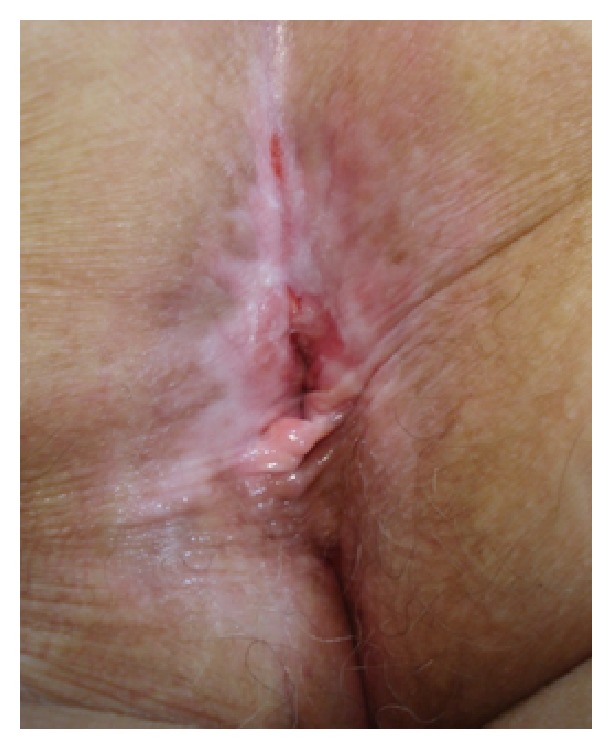
The perineal and perianal areas at 36 months postoperatively with a satisfactory appearance and no recurrence.

The entire wound was left open to heal by secondary intention. These conservative therapies went without complications. She is now in the 36th month of follow-up (every 6 months) and presents no residual lesions or recurrence. Furthermore, the functional results were excellent with no evidence of anal canal or incontinence, and the cosmetic results were satisfactory because moderate depigmentation and a little superficial atrophic scar remained in the treated area (Figure 3).

## 3. Discussion

BLT is a slow-growing, expansive, cauliflower-like, destructive lesion [6, 7] that occurs very rarely in female population [8]. The location is chiefly the vulva (90%) and an anorectal location is less frequent [9]. Most patients are adults and present with long-standing symptoms, a finding noted in our patient with a median duration of symptoms of 5 years [2, 10–13]. The main differential diagnosis includes hemorrhoids so the General Practitioner should know this rare tumour. Furthermore, it is important to note the psychological influence of the disease on the patient, whose feeling of shame permitted the lesion to evolve to this point, despite pain and bleeding [14].

The exact origin and etiology are not completely understood yet. Common opinion is that it is a viral infection associated mostly with the presence of HPV types 6 and 11 and very rarely with the presence of HPV types 16 and 18 [8]. The pathogenesis of HPV infection is undoubtedly influenced by the host's immune lymphocytes and natural killer cells, whose activity is boosted by interferons [7]. Immunosuppression is a risk factor for the rapid growing of condylomas and their malignant transformation [15, 16]. The patient's immunological status must be checked including screening test serology for STDs (HIV, syphilis, hepatitis B virus (HBV), and hepatitis C virus (HVC)) [17]. Our patient has an infection with HPV serotype 11 and is not currently immunosuppressed, although she underwent radiotherapy and chemotherapy in the past.

The locoregional extension must be carefully assessed to establish the therapeutic strategy. In respect to this, abdominal and pelvic magnetic resonance imaging is useful [7].

The BLT diagnosis can be difficult due to the lack of malignant cytological characteristics, especially if the biopsy includes only the surface epithelium. Therefore, a large and deep biopsy with carefully performed sections and a detailed histopathological examination is required [14].

Treatment of BLT can be classified into three types: topical therapy, tumour removal (surgery), and immunotherapy [18–20]. However, no gold standard currently exists for treating this rare disease. Treatment depends on the size of the lesion, how deep it is, its location, previous treatment, and also the physician's experience and skills [20]. In severe invasive cases, a surgical excision often plays a central role in the treatment of the BLT; however, surgical excision needs expert surgical technique, sophisticated anesthesia, and plastic reconstruction [21] and cannot avoid recurrence in more than 50% of patients [13, 18]. Furthermore, poor wound healing, fecal contamination of the operative site, fistulization, perineal abscesses, ulceration, difficulty in controlling hemorrhages, and extensive removal of soft tissue contribute to considerable postoperative morbidity and mortality [7, 13, 18, 22]. In addition, the emotional trauma associated with vulvar surgery, particularly in younger women, is considerable, and there is the risk of development of a phychosexual condition [23]. In light of these findings, cryotherapy and electrosurgery can be considered a good choice of treatment in noninvasive cases with a good response, and these techniques are considered minimally invasive surgery. In our case the CT scan excluded local invasion. It is very important to destroy the tissue to a depth of at least 5 to 8 mm because BLT infiltrates deeply into the underlying stroma [24]. In this case, the treatment was applied conveniently and economically with a bloodless field and minimal destruction to surrounding tissue. Unfortunately, the patient has a moderate depigmentation and a little superficial atrophic scar.

Chemotherapy and radiation therapy should be used only in case of disease recurrence because their effectiveness has not been fully documented [25].

A close follow-up is recommended in order to detect recurrence in an early phase. To date, there is no data in the literature in regard to the duration of follow-up. It seems that the recurrences are more frequent in the first months after surgery [26]. Currently our patient has been followed up every six months.

In conclusion, BLT is an infrequent and likely virulent infection with a challenging treatment because this tumour is characteristically locally very aggressive.

Therefore, prospective studies are necessary to further define the nature and treatment of this very rare disease.

Minimally invasive techniques such as cryotherapy and electrosurgery can be safer and more cost-effective than surgery. However, these techniques can only be applied in noninvasive cases.

As in a significant percentage of patients, the first manifestations of BLT are a growing mass with pain and bleeding; therefore the General Practitioner can play a key role in diagnosis.

When there is a BLT, a multidisciplinary team including dermatology should be involved in treatment and follow-up.

Finally, careful observation over a prolonged period of time is essential in order to detect recurrences and control potential complications related to the treatment.
